# Supplementary material for: Molecular signatures of cortical expansion in the human foetal brain
Source: Nat Commun. 2024 Nov 8;15:9685. doi: 10.1038/s41467-024-54034-2 (PMC11549424; doi:10.1038/s41467-024-54034-2)
Supplement: Supplementary file 1 — Supplementary Information [file 41467_2024_54034_MOESM1_ESM.docx]

Molecular signatures of cortical expansion in the human fetal brain

G. Ball, S. Oldham, V. Kyriakopoulou, L. Z. J. Williams, V. Karolis, A. Price, J. Hutter, M.L. Seal, A. Alexander-Bloch, J.V. Hajnal, A.D. Edwards, E.C. Robinson, J. Seidlitz

**SUPPLEMENTAL MATERIALS**

Supplemental figures

**Figure S1: Lightbox of reference atlas sections**

**Figure S2: Model performance over hyperparameters**

**Figure S3: Shape reference for affine registration**

**Figure S4.** $\mu$**Brain volume, atlas, cortical surfaces and microarray data**

**Figure S5: All ISH reconstructions.**

**Figure S6: PCA analysis of each brain specimen**

**Figure S7: Correlation between PC1 and age-related changes in expression in each tissue**

**Figure S8: Correlation between age-related gene expression changes across tissue**

**Figure S9: Developmental tissue enrichment of ZRT and non-ZRT genes**

**Figure S10: Cell type enrichment across tissue zones.**

**Figure S11: Gestational age distribution of fetal MRI scans (n=195) included in analysis.**

**Figure S12: Comparison of within- and across-subject estimates of cortical expansion.**

**Figure S13: Differential expression over tissue zones in ZRT genes.**

**Figure S14: Tissue and cell expression of ZRT_neo_ genes at 15 PCW.**

**Figure S15: SATB2 ISH across cortical regions.**

**Figure S16: Expression of OPC markers in the intermediate zone.**

**Figure S17: OLIG1 expression in prenatal bulk tissue mRNA data.**

**Figure S18: Chromatin accessibility near to ZRT genes in the fetal brain.**

**Figure S20: Image registration pipeline and template generation.**

**Figure S21: Cortical surface area scaling after removing repeated scans.**

**Figure S22: Proportion of vertex outliers in surface area data**

Supplemental references

# Supplemental figures


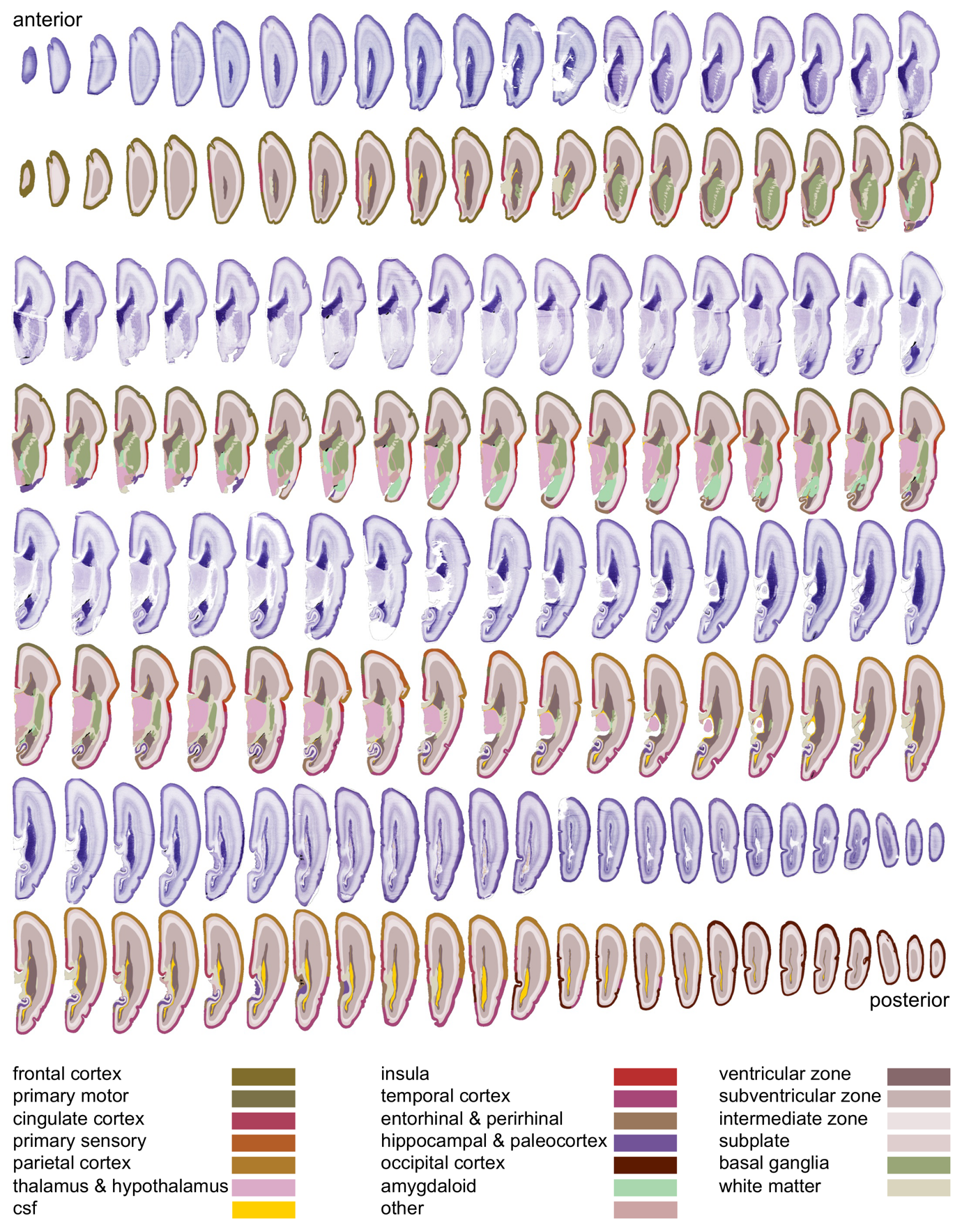


#### Figure S1: Lightbox of reference atlas sections

Serial Nissl-stained sections and corresponding anatomical annotations used to construct the $\mu$Brain atlas. Annotations represent a simplified label set (brain-labels) based on the hierarchical ontology of the BrainSpan reference atlas (**Supplemental Data S2**).


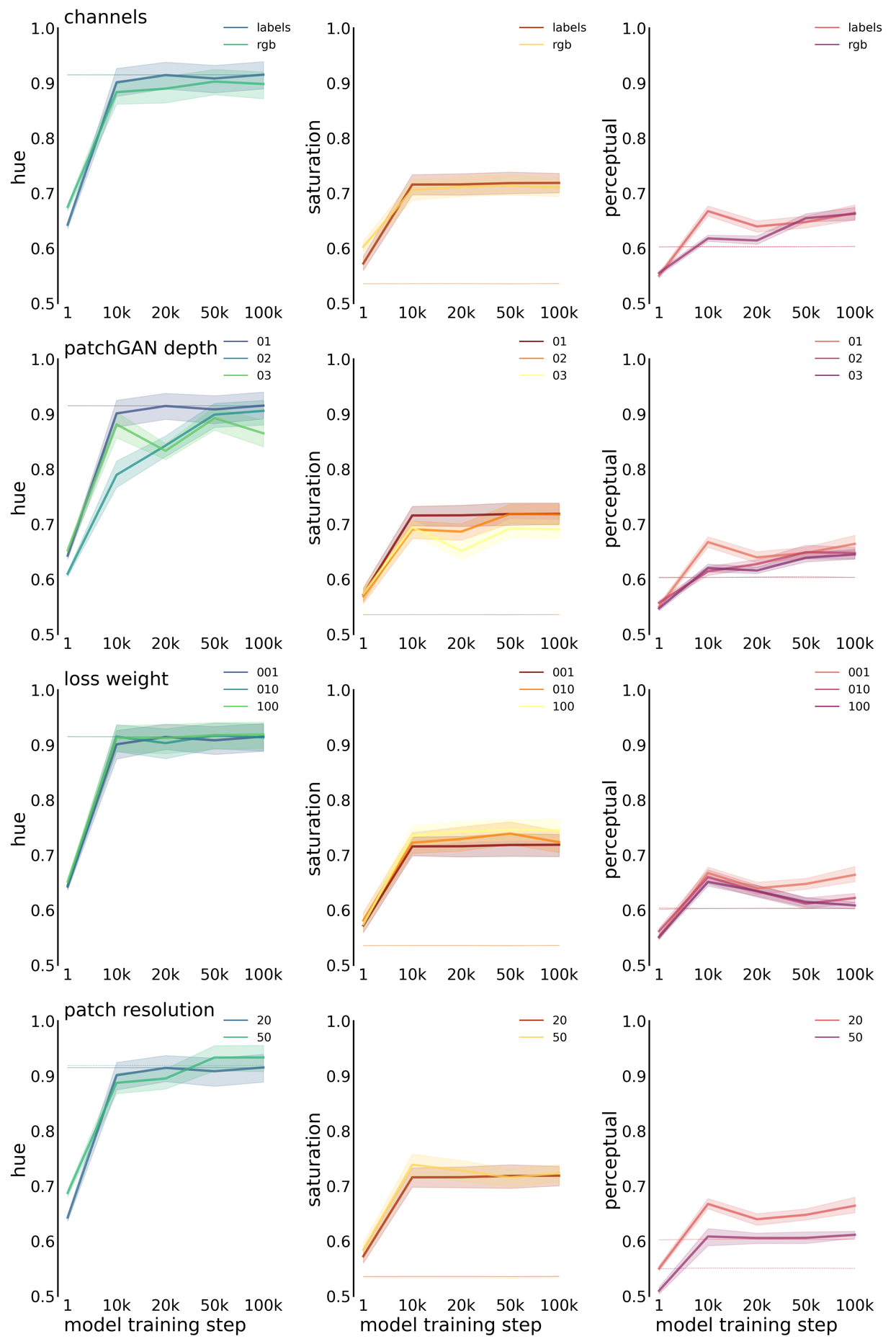


Figure S2: Model performance over hyperparameters

Mean similarity of hue (left) and saturation (middle) of patch predictions compared to ground truth during model training with different parameters (patch resolution in $\mu$m; loss weight; number of *patchGAN* layers and input channels). Shaded areas show 95% C.I. across samples. Perceptual similarity (right) was calculated based on the similarity of outputs from a pretrained VGG19 model. Baseline measures of similarity were generated by randomising pixels within each patch (dashed lines). Source data are provided as a Source Data file.


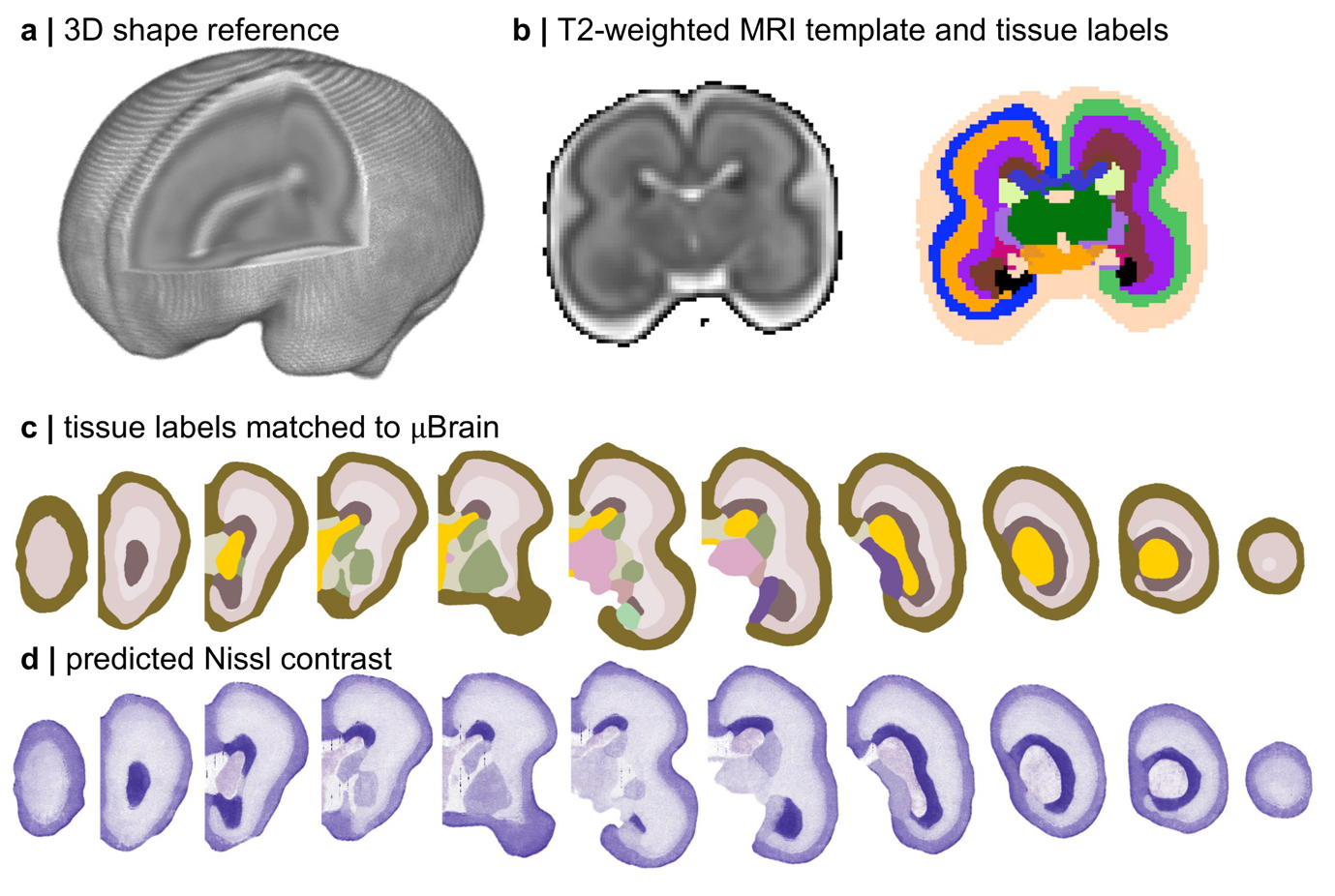


#### Figure S3: Shape reference for affine registration

a. A fetal brain atlas at 22 gestational weeks was used as a shape reference for affine registration. b. manual tissue labels were matched to $\mu$Brain anatomical labels (c) and used to generate synthetic ‘Nissl-contrast’ sections (d) to act as a target for registration.


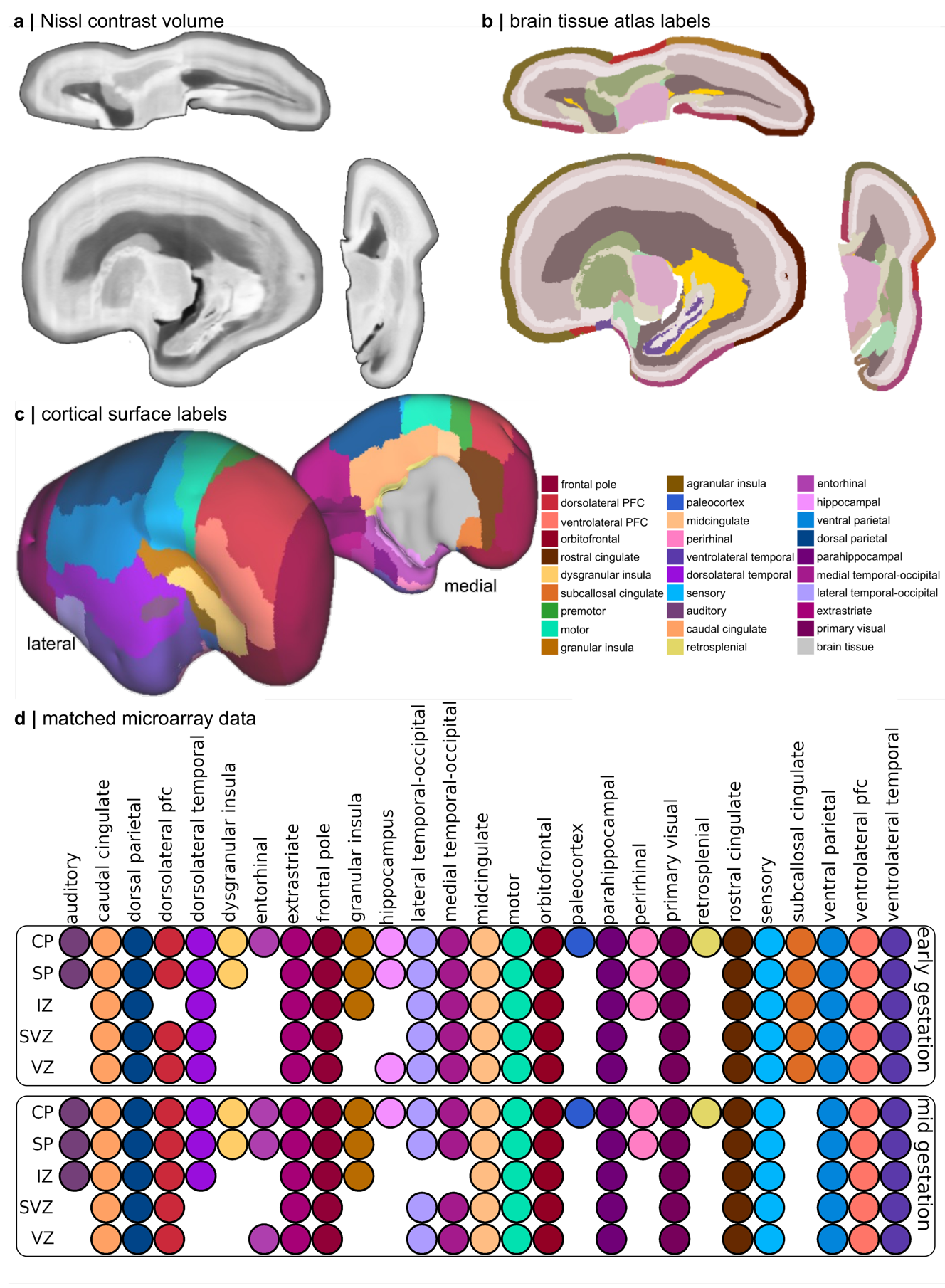


#### Figure S4. $\boldsymbol{\mu}$Brain volume, atlas, cortical surfaces and microarray data

a. 3D volumetric reconstruction of histological data. b.corresponding anatomical atlas labels (brain-labels; **Supplemental Data S2**). Coloured as in **Figure S1**. c. lateral and medial cortical surface reconstruction labelled by cortical area (cortex-labels; **Supplemental Data S2**). d. available LMD microarray data matched to cortical labels at early and mid gestational timepoints (15/16PCW and 21PCW, respectively) for each of five tissue zones. CP: cortical plate; SP: subplate; IZ, intermediate zone; SVZ: subventricular zone; VZ: ventricular zone


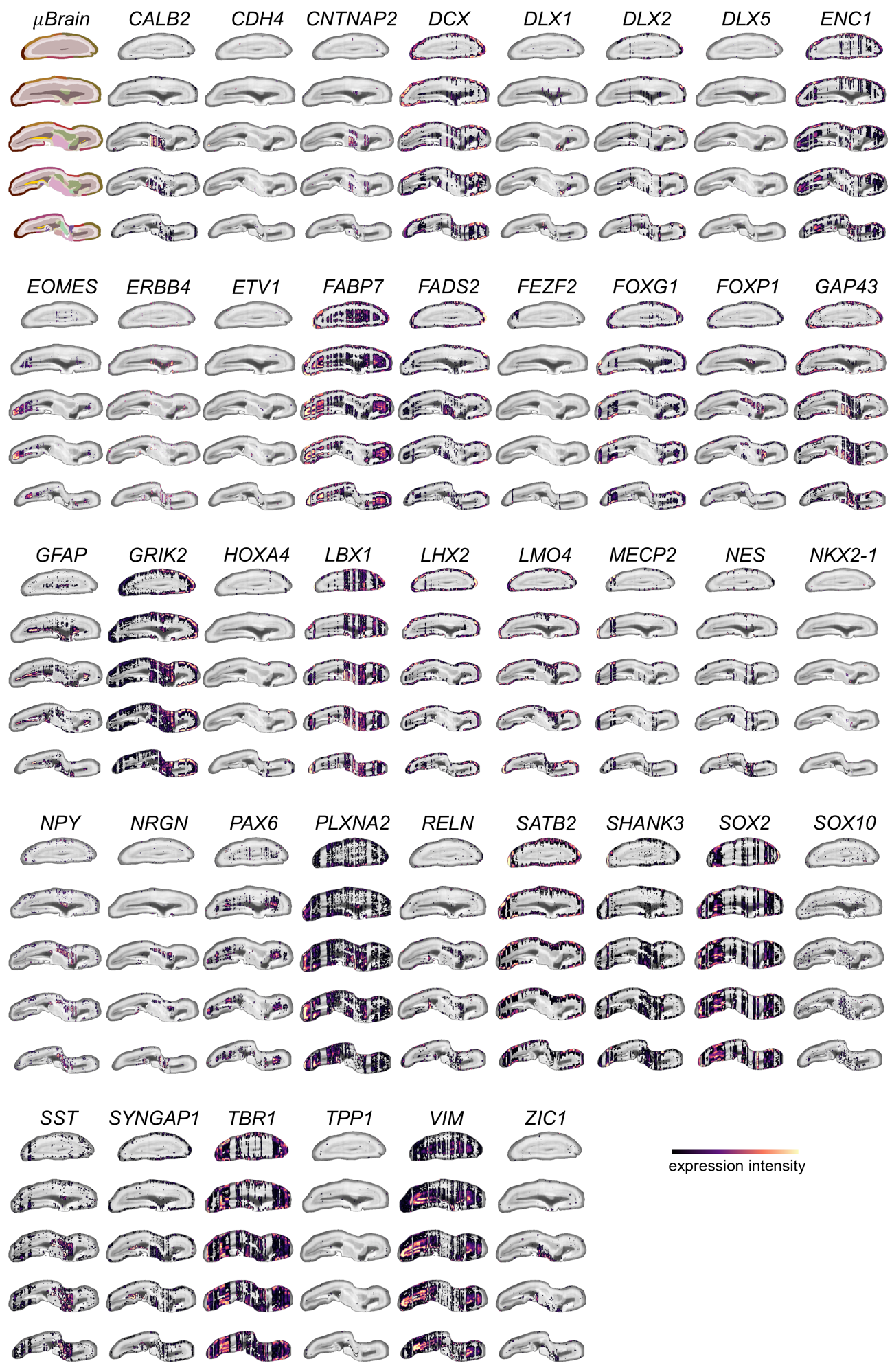


#### Figure S5: All ISH reconstructions.

Axial slices from partial 3D reconstruction of ISH expression data from n=41 neurodevelopmental genes.


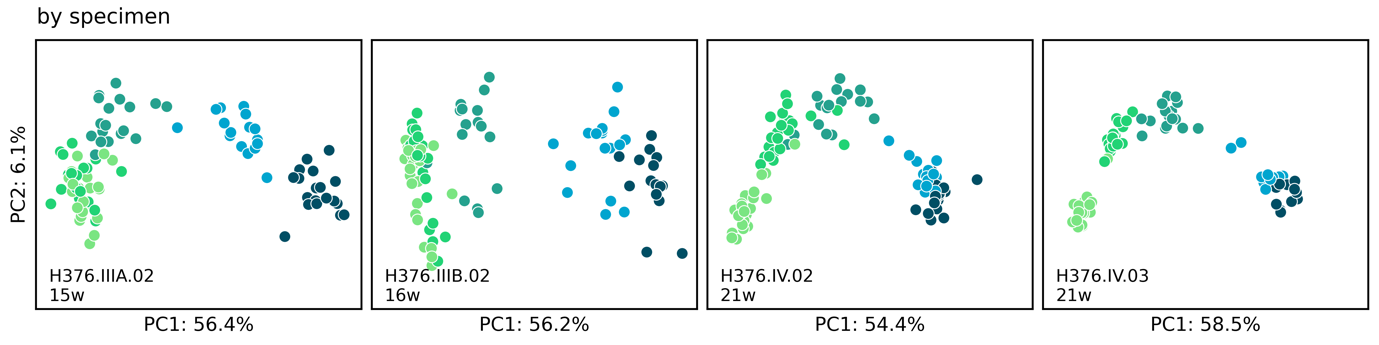


#### Figure S6: PCA analysis of each brain specimen

PCA was performed separately on each specimen’s microarray data. The first two components are shown for each specimen. Colours represent tissue layers as in Fig 2a. Source data are provided as a Source Data file.


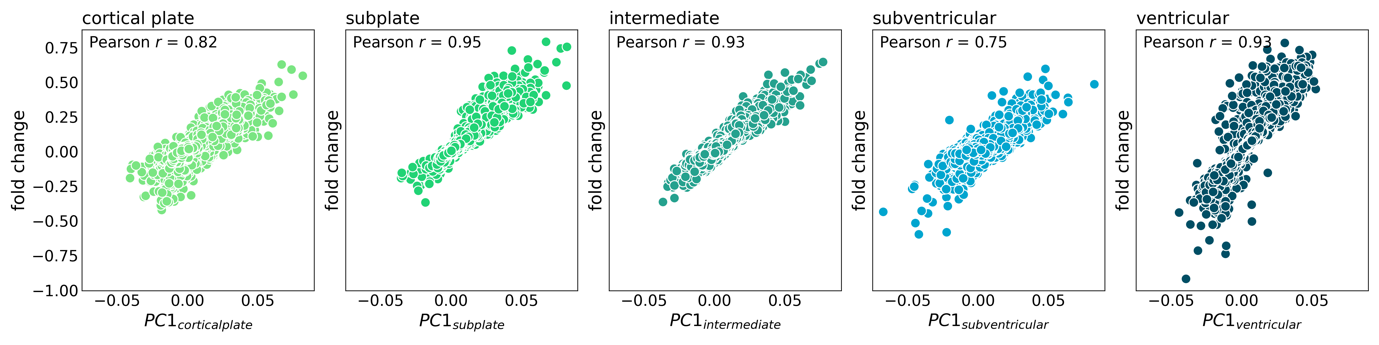


#### Figure S7: Correlation between PC1 and age-related changes in expression in each tissue

For each tissue zone, we calculated the correlation between age-related changes (between 15 and 21PCW) and the first principal component derived from all samples within each tissue zone. Age related change explained the majority of variance in each tissue zone. Source data are provided as a Source Data file.


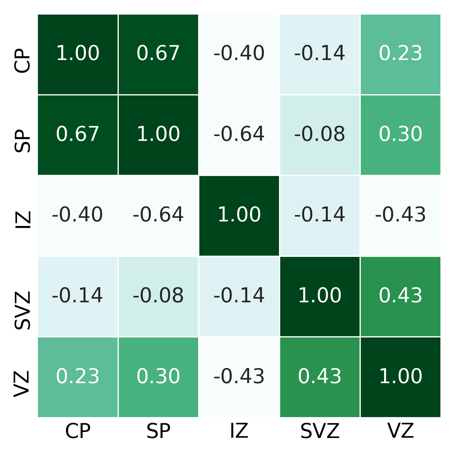


#### Figure S8: Correlation between age-related gene expression changes across tissue

Correlation between age-related changes in each gene across tissue zones. Age-related changes in gene expression were most similar between neighbouring tissue zones.


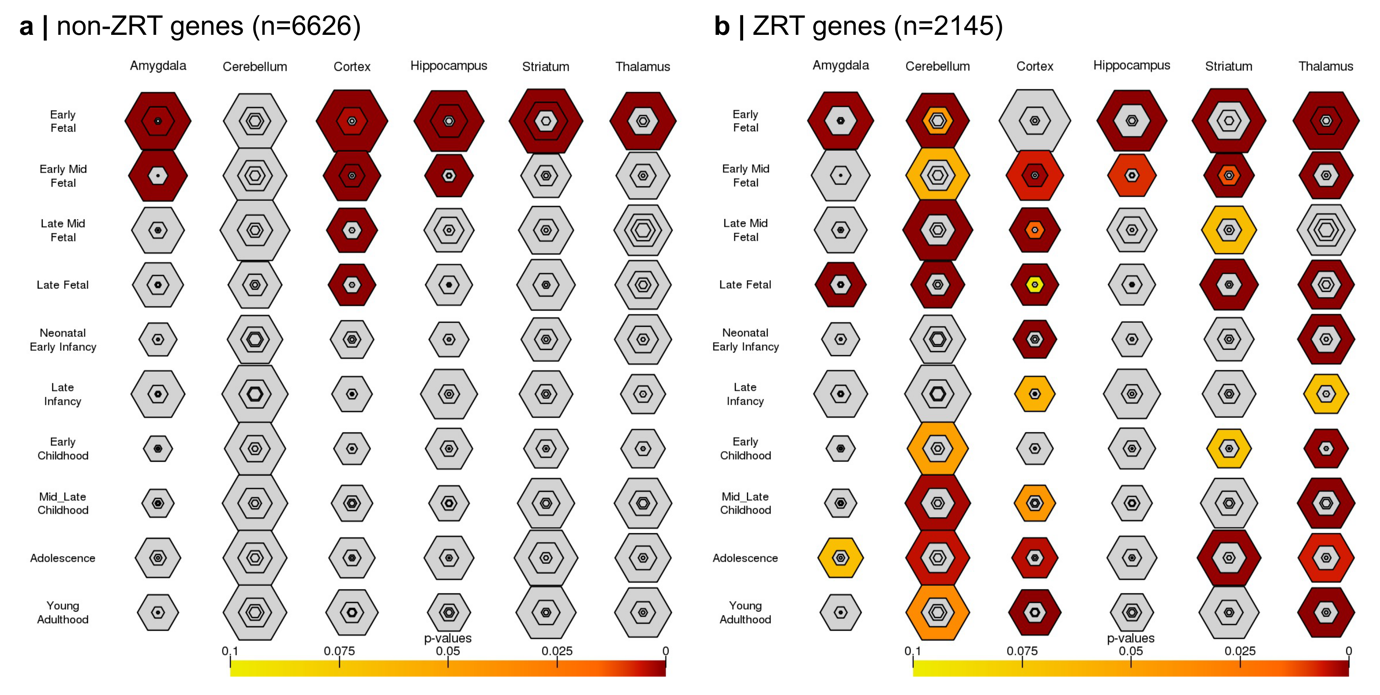


#### Figure S9: Developmental tissue enrichment of ZRT and non-ZRT genes

Output from Cell-Specific Enrichment Analysis^1^ of ZRT and non-ZRT genes. Significant, cell-specific enrichment of genes are illustrated by hexagon size and colour.

Figure S10: Cell type enrichment across tissue zones.

ZRT genes differentially expressed in each tissue zone (p<0.05 uncorr., one-sided hypergeometric test) were identified. Cell-type enrichment for each set of differentially-expressed genes are shown. Significant cell type enrichments are highlighted in black. UMAP plots show enriched cell clusters in each tissue zone. Cell cluster and type assignments are from Bhaduri et al.^2^ CR: Cajal-Retzius cells; IPC: intermediate progenitor cells; OPC: oligodendrocyte progenitor cells; RG: radial glia. Source data are provided as a Source Data file.


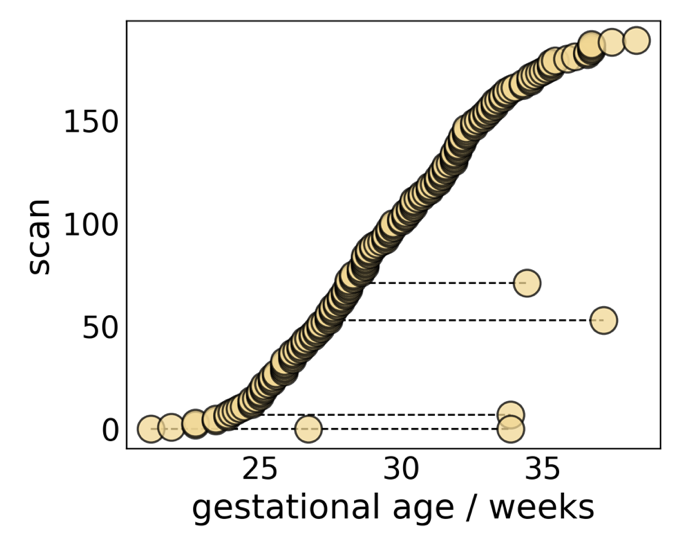


#### Figure S11: Gestational age distribution of fetal MRI scans (n=195) included in analysis.

Four fetuses were scanned more than once during gestation. Source data are provided as a Source Data file.


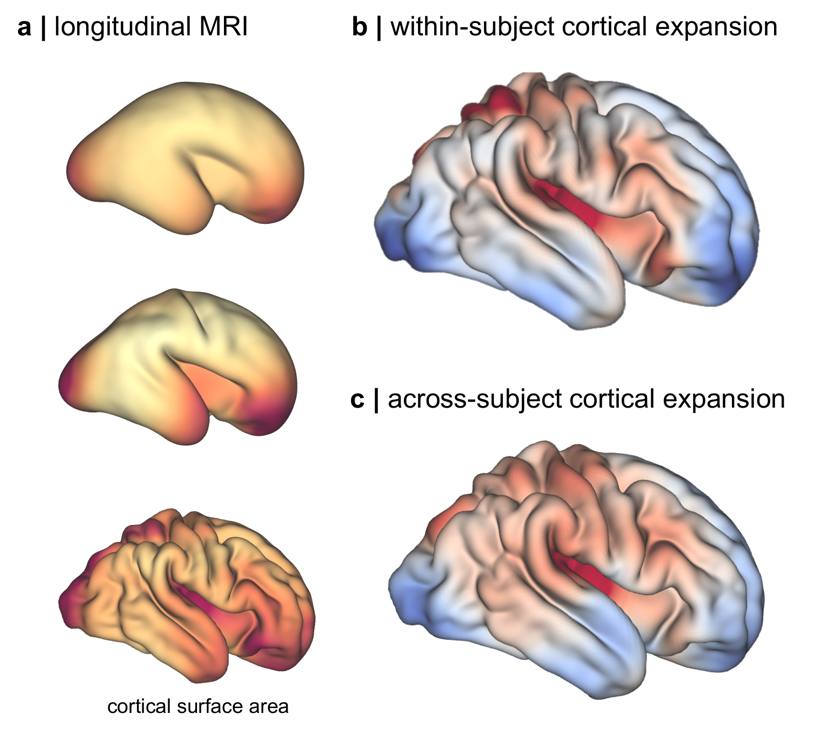


#### Figure S12: Comparison of within- and across-subject estimates of cortical expansion.

a. A single fetus was scanned three times during gestation After MRI processing, cortical surfaces were extracted from each scan and vertex area was calculated after resampling native topology onto the dHCP template surface. b. Differences in proportional area, calculated as the area at each vertex divided by total cortical surface area, between the earliest and latest timepoints. Warm colours indicate a preferential increase in regional surface area over time. c. Whole-group, cross-sectional expansion map from **Figure 3**.


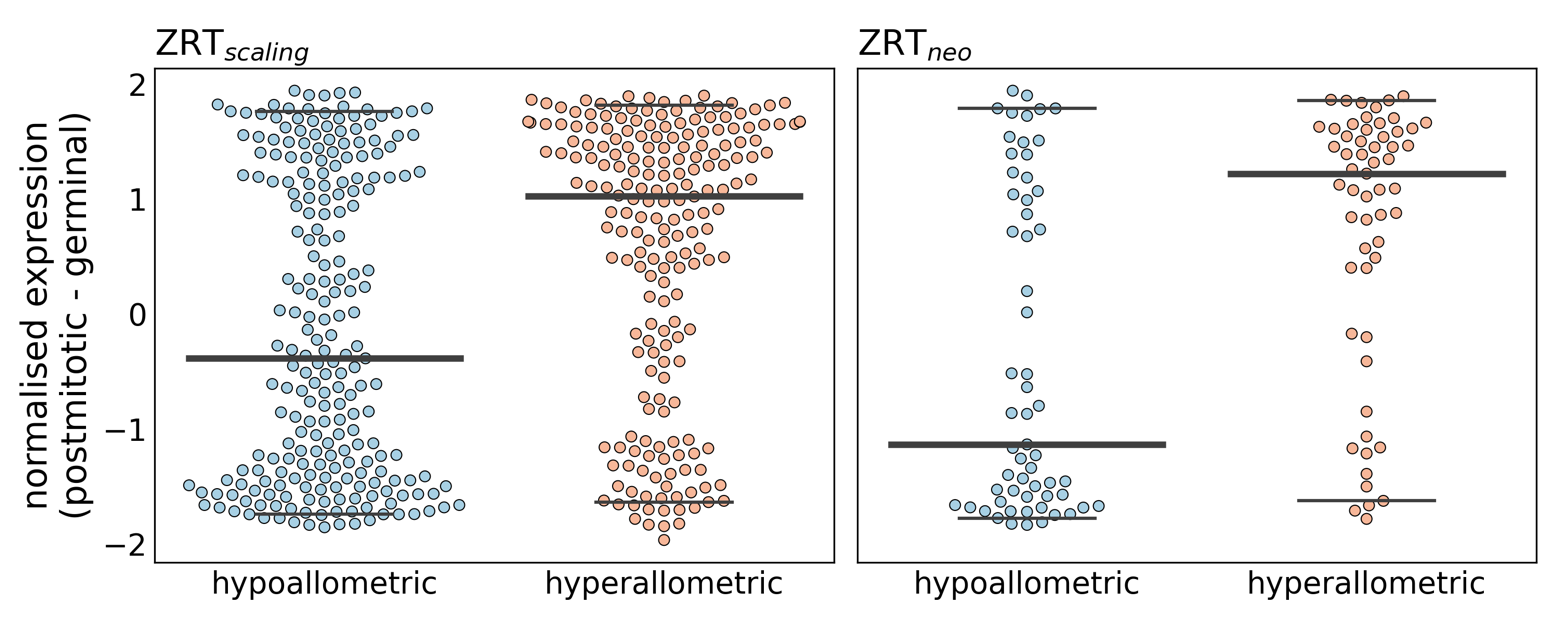


Figure S13: Differential expression over tissue zones in ZRT genes.

Plots show the mean difference in expression of hyper- and hypoallometric ZRT_scaling_ (left) and ZRT_neo_ (right) genes in postmitotic zones (CP, SP, IZ) and germinal zones (SVZ, VZ). Positive values indicate higher expression in postmitotic zones. Horizontal bars indicate median difference with 95% C.I. Source data are provided as a Source Data file.


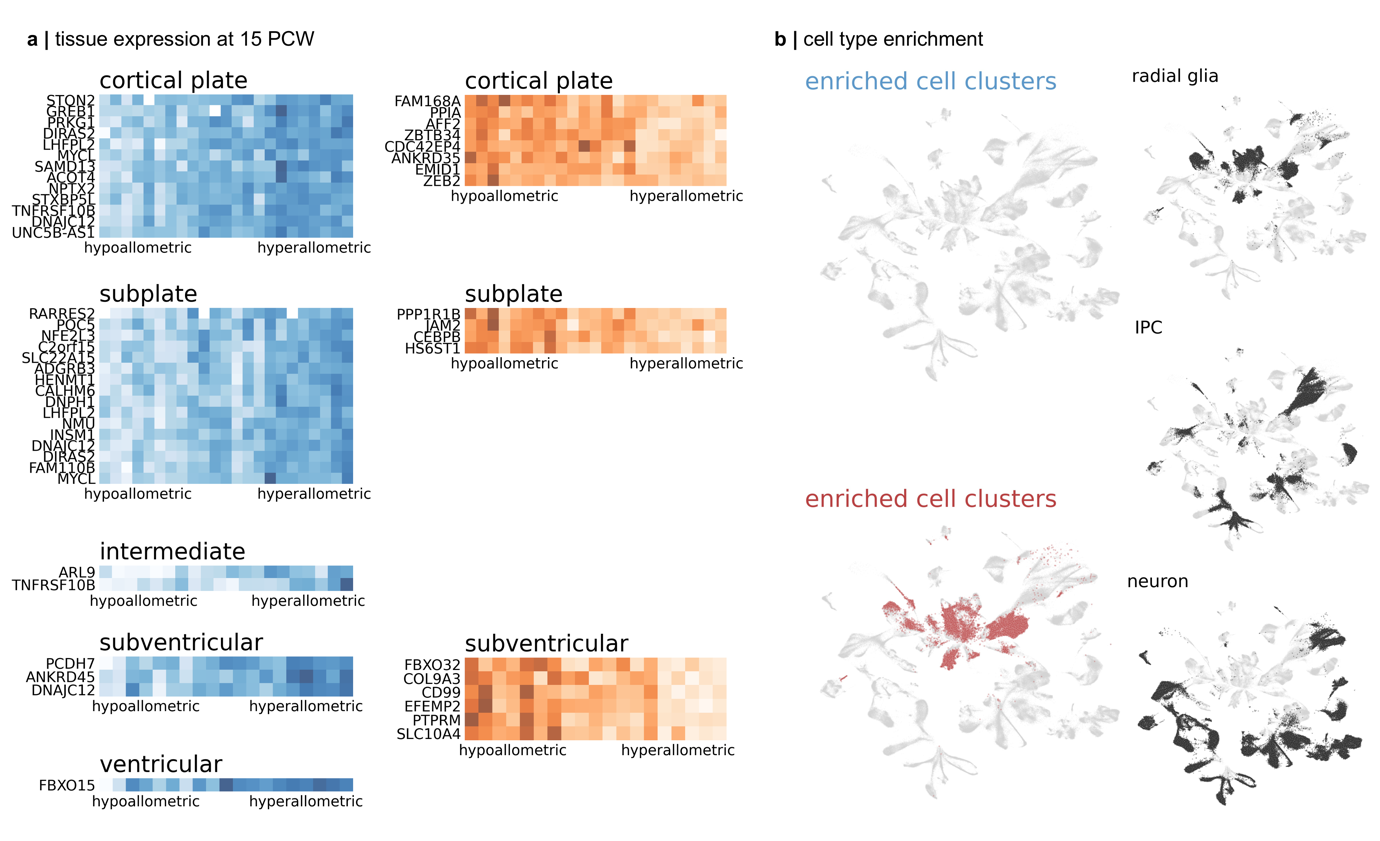


#### Figure S14: Tissue and cell expression of ZRT_neo_ genes at 15 PCW.

**a.** normalised (Z-score) expression profiles for genes correlated with areal scaling in each tissue zone at 15PCW. Negative associations (higher relative expression in hypoallometric regions) shown in blue, positive associations are in red. Lighter colours indicate higher relative expression. **b.** mid-gestation cell clusters^2^ significantly enriched for genes associated with areal scaling in at 15PCW. Territories of three canonical cell types are shown. Source data are provided as a Source Data file.


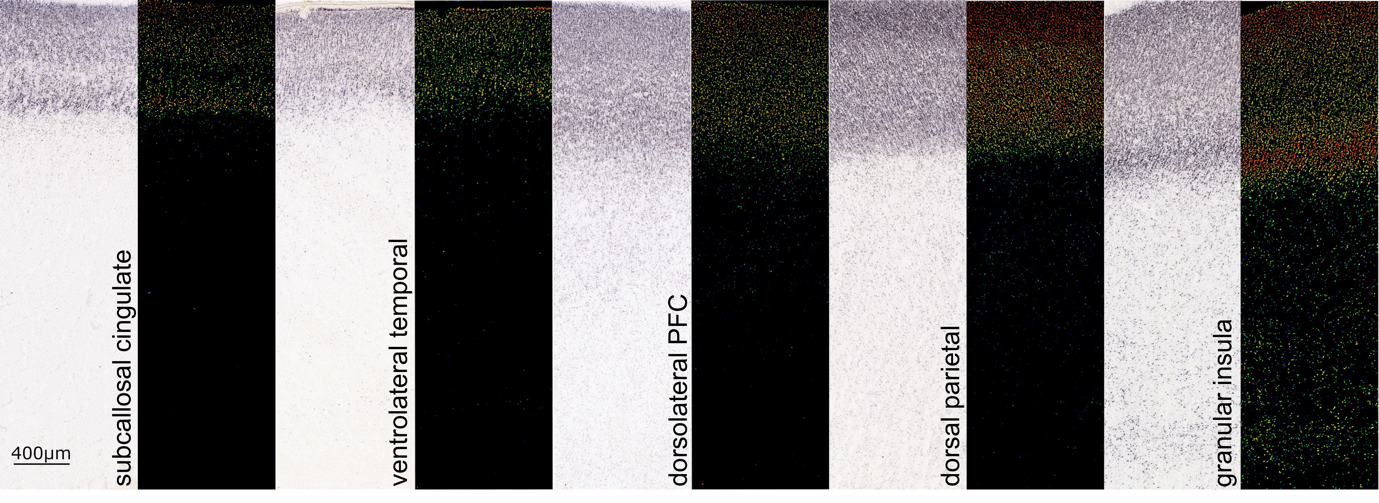


#### Figure S15: SATB2 ISH across cortical regions.

Example ISH staining of SATB2^+^ cells across the cortical anlage in five cortical regions.


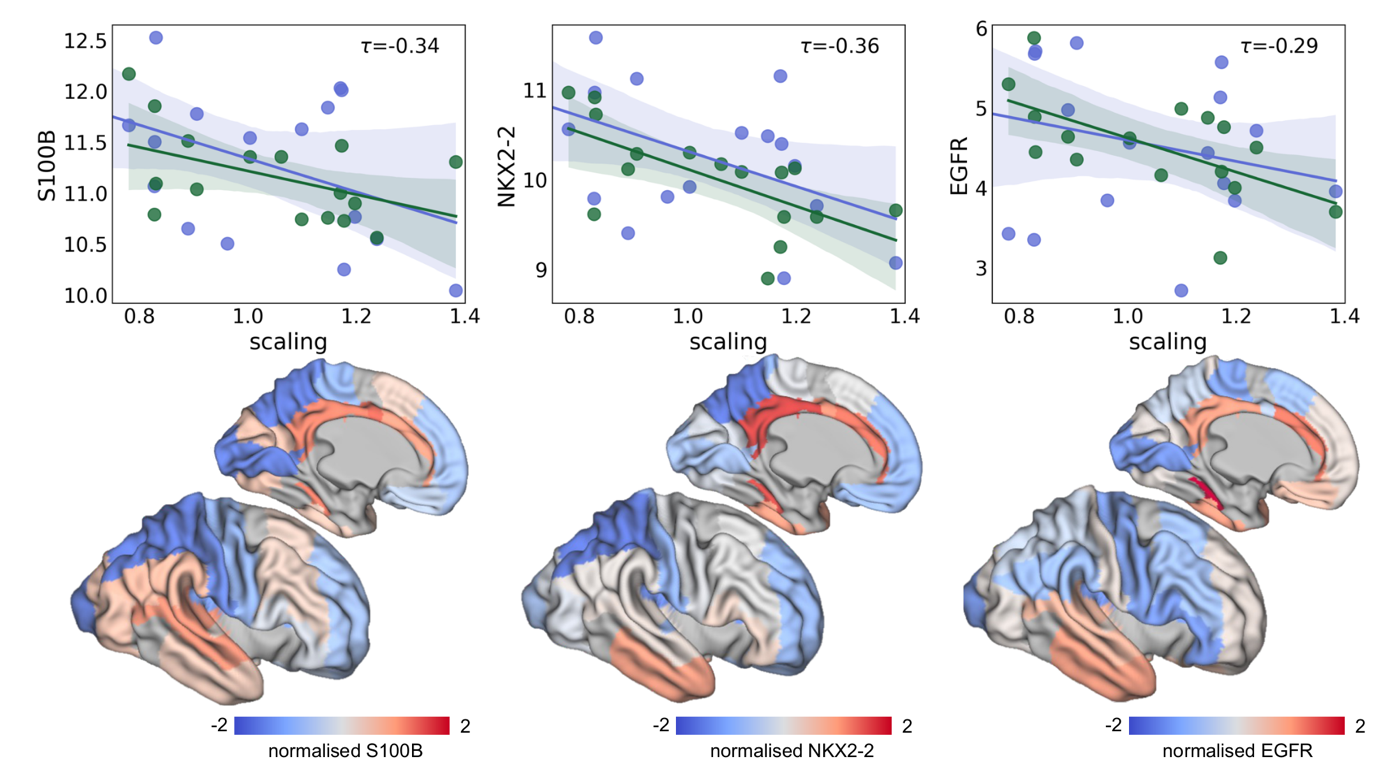


#### Figure S16: Expression of OPC markers in the intermediate zone.

Top: regional expression of oligodendrocyte-lineage markers (S100B, NKX2-2, EGFR) in the intermediate zone of two mid-gestation brains is associated with cortical expansion (scaling coefficient). Samples, coloured by specimen, are shown with linear regression and 95% C.I. Bottom: Mean normalised expression of each marker in the IZ calculated within each $\mu$Brain label and projected onto the 35w surface template. Source data are provided as a Source Data file.


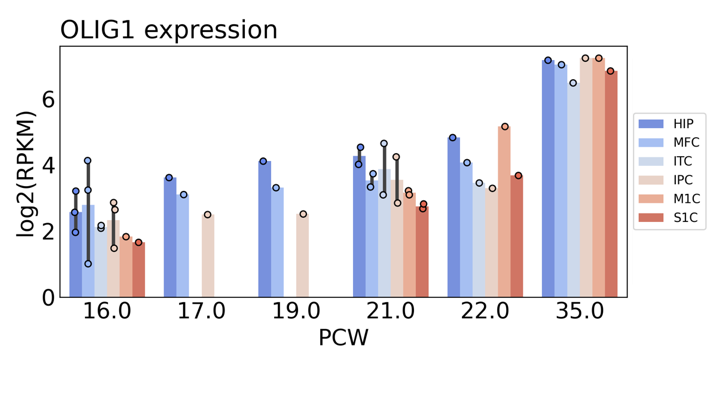


#### Figure S17: OLIG1 expression in prenatal bulk tissue mRNA data.

Regional estimates of OLIG1 expression were extracted from six cortical regions with differential allometric expansion (hypoallometric=blue; hyperallometric=red) for n=9 prenatal brain specimens aged 16 – 35PCW. HIP: hippocampus; MFC: medial frontal cortex; ITC: inferior temporal cortex; IPC: inferior parietal cortex; M1C: primary motor cortex; S1C: primary sensory cortex. Source data are provided as a Source Data file.


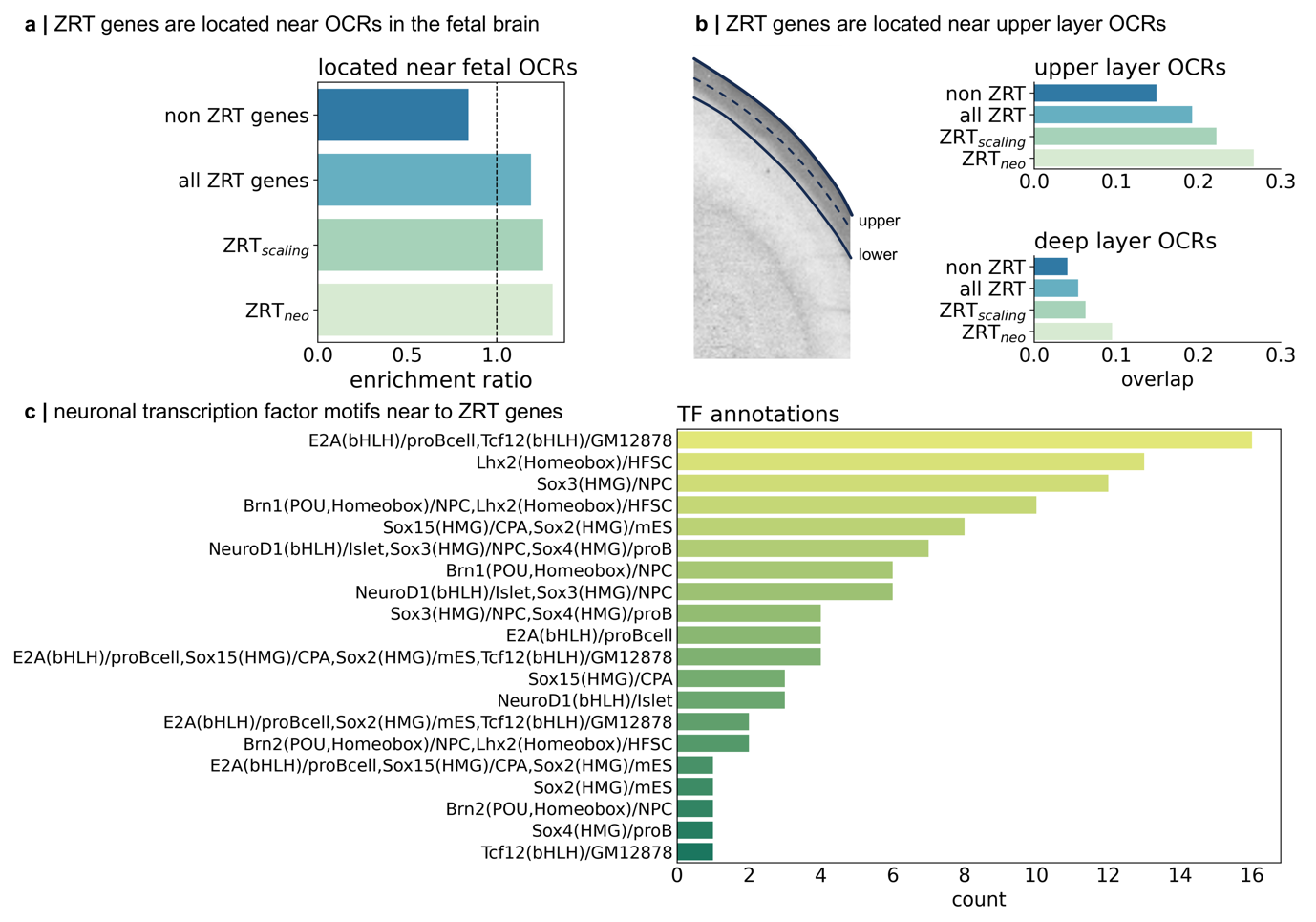


#### Figure S18: Chromatin accessibility near to ZRT genes in the fetal brain.

**a**. enrichment of different ZRT genesets in all genes located near to OCRs in the prenatal brain. **b.** enrichment in laminar-specific OCRs. **c.** number of transcription factor motif annotations located near to ZRT genes. Source data are provided as a Source Data file.


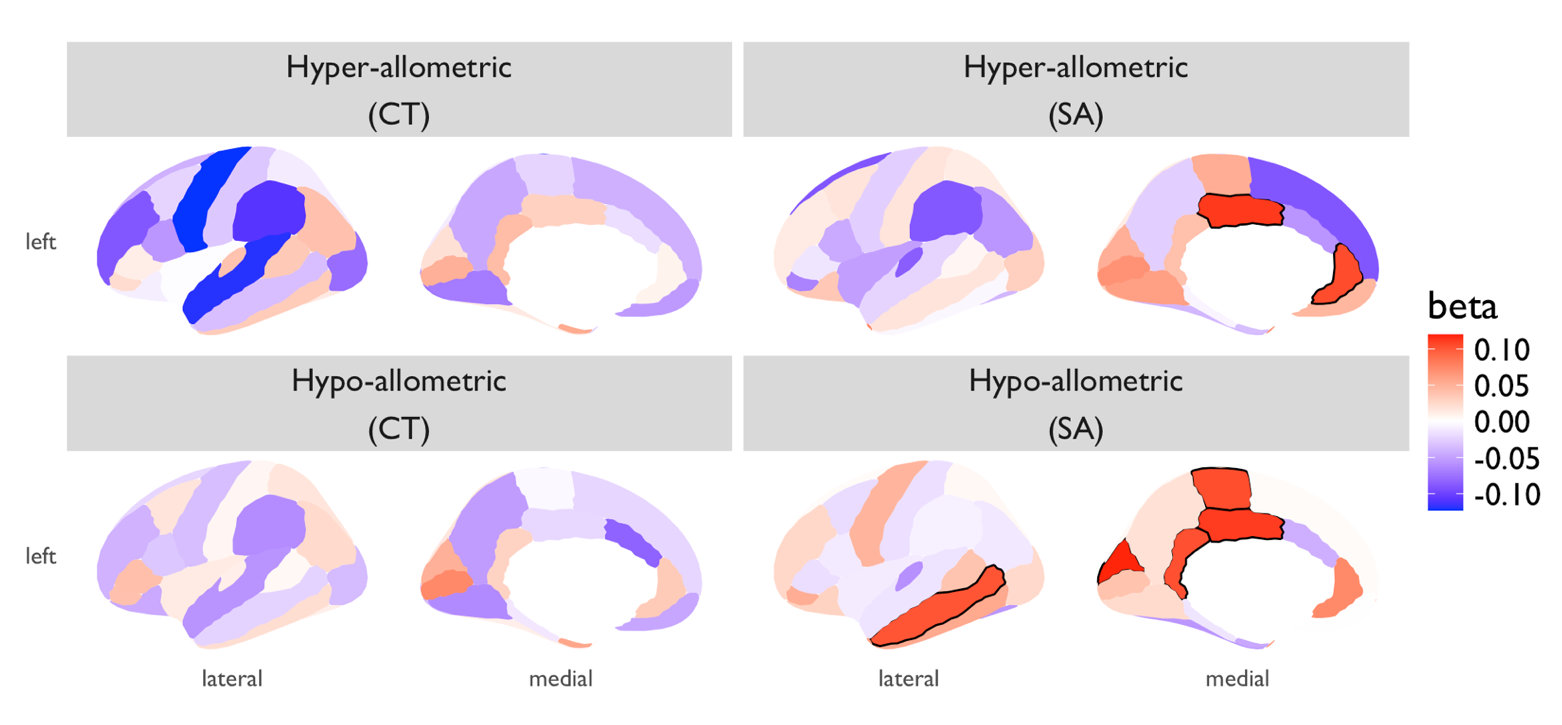

Figure S19. Bilateral cortical thickness (CT) and surface area (SA) GWAS enrichment results of hyper- and hypo-allometric scaling gene sets.

Beta coefficients for gene-set enrichment analyses performed with MAGMA are plotted on cortical surfaces (left hemisphere shown just for visualization). Black outlines denote significant enrichment (p < 0.05, uncorrected).

####
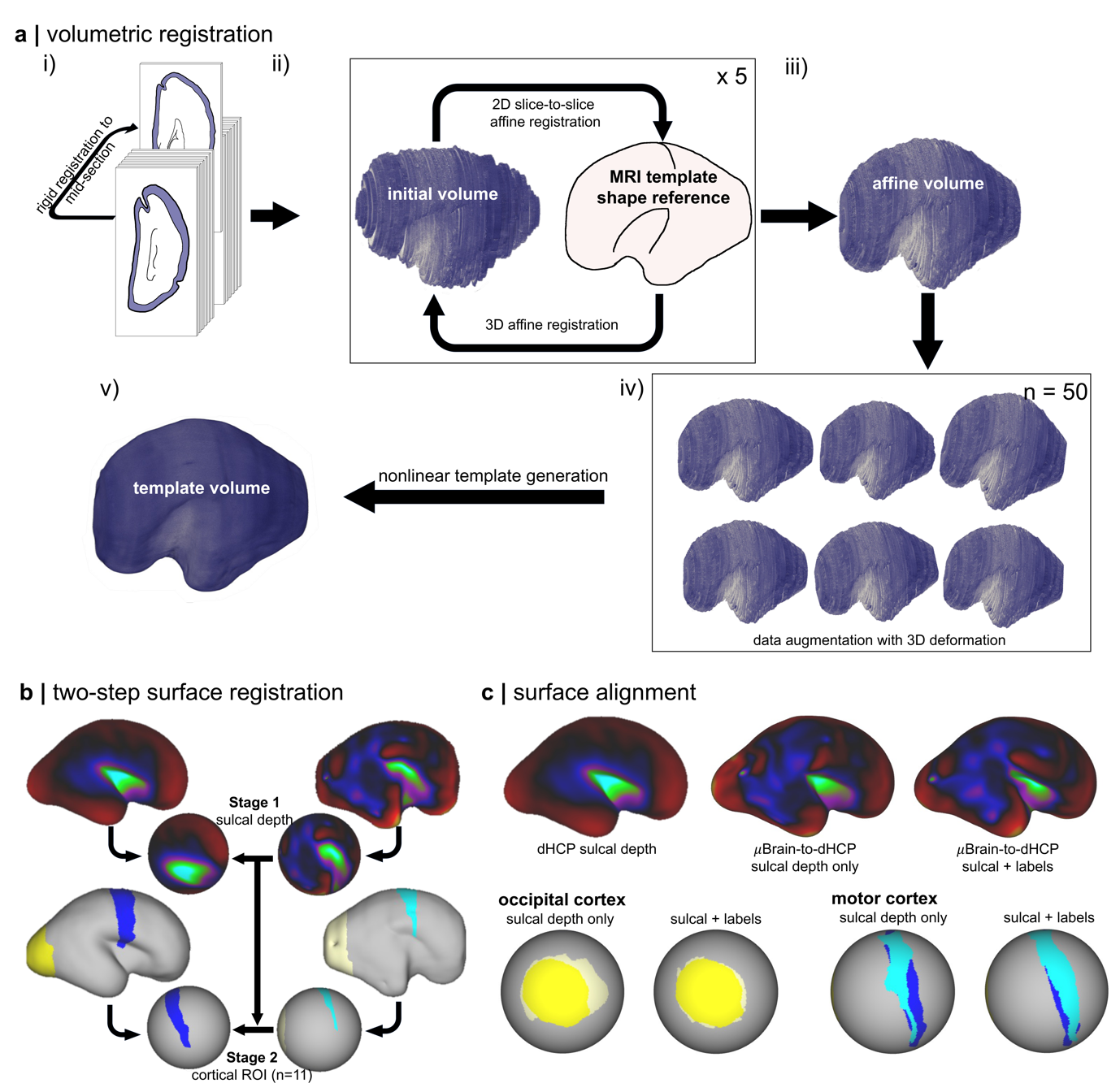
Figure S20: Image registration pipeline and template generation.

**a**. clockwise: i) Repaired Nissl sections were first aligned to the mid-section using a graph-based rigid registration algorithm and stacked into a 3D volume. ii) An MRI-based anatomical template was used as a shape reference to guide affine alignment between sections. After transforming each slice of the template to a Nissl-like contrast using the trained *pix2pix* model (**Figure S3**), we aligned the reference to the Nissl volume using a 3D affine registration. The aligned volume was resliced and 2D section-to-slice registration to the shape reference performed for each Nissl section. This process was repeated for 5 iterations to form an ‘affine volume’ (iii). iv)We employed a data augmentation to induce 3D deformations in the affine volume and create a population (n=50) of alternative representations of the cerebral volume, resampled to 150$\mu$m resolution. For each volume, each section was registered to its neighbours using nonlinear registration. Registrations were performed slice-by-slice, once forward and once backwards along the volume for a total of 3 iterations. v) All 50 volumes were co-registered and averaged to create a final 3D template using symmetric nonlinear registration. **b**. A two-step registration process was used to align the $\mu$Brain cortical surface to the dHCP fetal template. Nonlinear surface registrations were performed between spherical representations of each surface using MSM driven by sulcal depth (Stage 1). This alignment was used to initialise a second registration driven by a set of matched cortical regions-of-interest (n=11; Stage 2). Only the occipital (yellow) and motor (blue) regions are shown for simplicity. Each cortical region was passed to the algorithm as a separate metric with alignment jointly optimised over all regions. **c**. cortical surface alignment after Stage 1 (sulcal depth only) and Stage 2 (sulcal depth + cortical labels). Top row shows sulcal depth maps on the fetal surface. Bottom rows shows alignment of occipital and motor regions on the spherical surface.


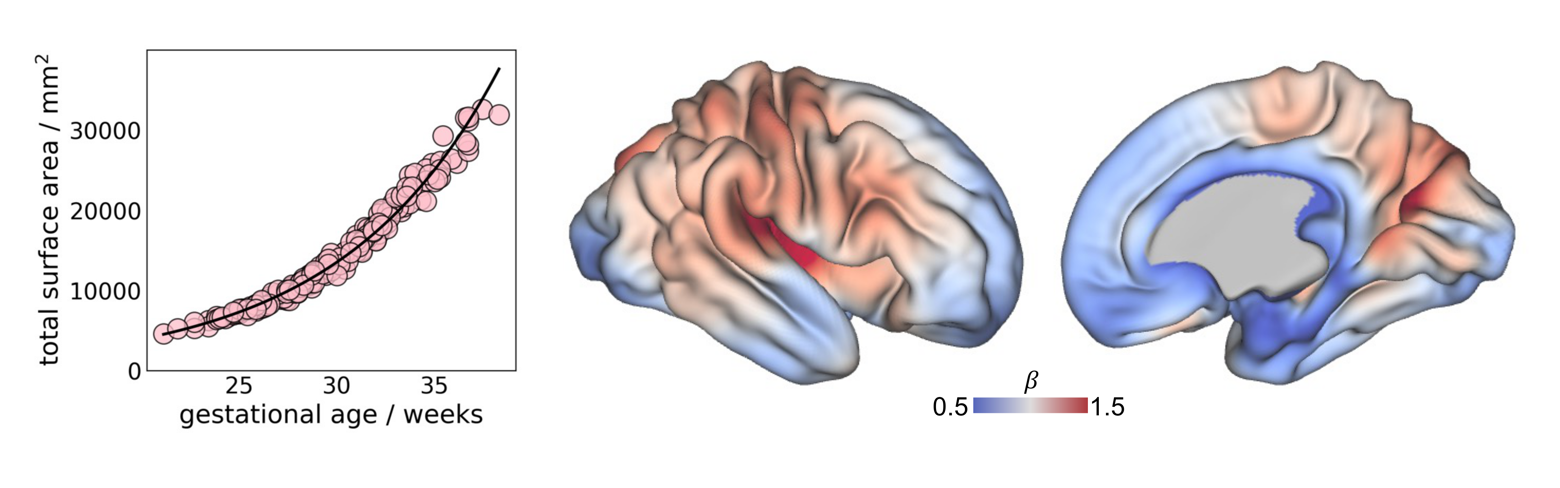


#### Figure S21: Cortical surface area scaling after removing repeated scans.

Source data are provided as a Source Data file.

**
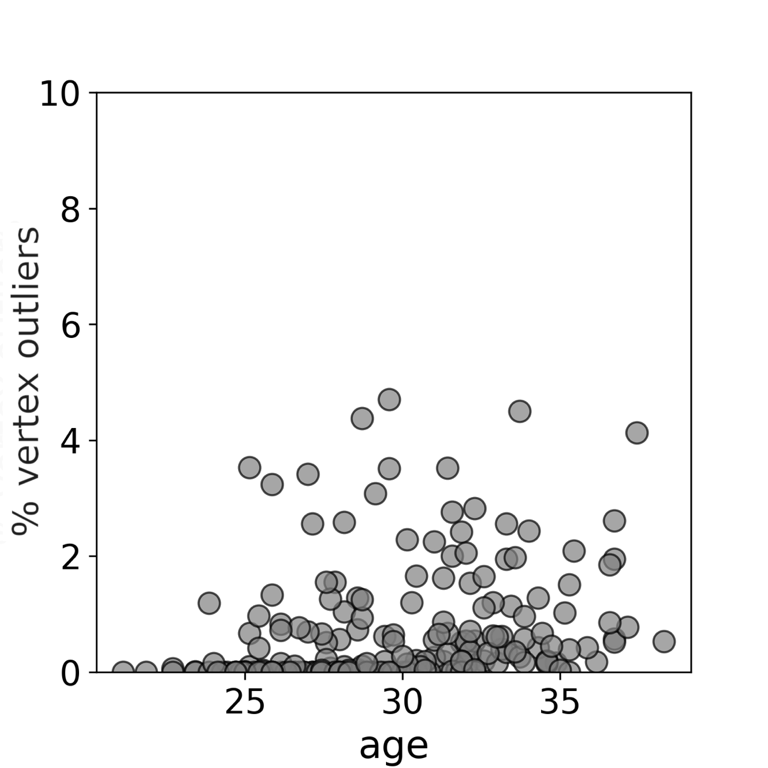
**

#### Figure S22: Proportion of vertex outliers in surface area data

Source data are provided as a Source Data file.

## Supplemental references

1. Dougherty, J. D., Schmidt, E. F., Nakajima, M. & Heintz, N. Analytical approaches to RNA profiling data for the identification of genes enriched in specific cells. *Nucleic Acids Res.* **38**, 4218–4230 (2010).

2. Bhaduri, A. *et al.* An atlas of cortical arealization identifies dynamic molecular signatures. *Nature* **598**, 200–204 (2021).
