# Supplementary material for: Molecular signatures of cortical expansion in the human foetal brain
Source: Nat Commun. 2024 Nov 8;15:9685. doi: 10.1038/s41467-024-54034-2 (PMC11549424; doi:10.1038/s41467-024-54034-2)
Supplement: Supplementary file 2 — Description of Additional Supplementary Files [file 41467_2024_54034_MOESM2_ESM.pdf]

Description of Additional Supplemental Files

Supplemental Data S1: Reference atlas section numbers and approximate positions

Supplemental Data S2: Anatomical groupings for each label set

Supplemental Data S3: In situ hybridisation data available for volumetric reconstruction

Supplemental Data S4: Anatomical groupings for LMD microarray data

Supplemental Data S5: Cell cluster enrichment of genes increasing (up) or decreasing (down) between 15 and 21PCW

Supplemental Data S6: Effects of age, region and tissue layer for each gene (n=8771)

Supplemental Data S7: ZRT cell type and cell cluster enrichment

Supplemental Data S8: ZRT geneset enrichment from WebGestalt

Supplemental Data S9: ZRT gene identities

Supplemental Data S10: ZRT associations with cortical and neocortical scaling

Supplemental Data S11: Cell type enrichment of ZRT_neo genes at 15 and 21PCW

Supplemental Data S12: Enrichment of ZRT genes near to open chromatin regions (OCR)
