# Supplementary material for: Molecular signatures of cortical expansion in the human foetal brain
Source: Nat Commun. 2024 Nov 8;15:9685. doi: 10.1038/s41467-024-54034-2 (PMC11549424; doi:10.1038/s41467-024-54034-2)
Supplement: Supplementary file 4 — Reporting Summary [file 41467_2024_54034_MOESM4_ESM.pdf]

nature portfolio

Corresponding author(s): Ball

Last updated by author(s): Mar 15, 2024

Reporting Summary

Nature Portfolio wishes to improve the reproducibility of the work that we publish. This form provides structure for consistency and transparency in reporting. For further information on Nature Portfolio policies, see our Editorial Policies and the Editorial Policy Checklist.

Statistics

For all statistical analyses, confirm that the following items are present in the figure legend, table legend, main text, or Methods section.

n/a	Confirmed	
☐	☒	The exact sample size (n) for each experimental group/condition, given as a discrete number and unit of measurement
☐	☒	A statement on whether measurements were taken from distinct samples or whether the same sample was measured repeatedly
☐	☒	The statistical test(s) used AND whether they are one- or two-sided<br>*Only common tests should be described solely by name; describe more complex techniques in the Methods section.*
☐	☒	A description of all covariates tested
☐	☒	A description of any assumptions or corrections, such as tests of normality and adjustment for multiple comparisons
☐	☒	A full description of the statistical parameters including central tendency (e.g. means) or other basic estimates (e.g. regression coefficient) AND variation (e.g. standard deviation) or associated estimates of uncertainty (e.g. confidence intervals)
☐	☒	For null hypothesis testing, the test statistic (e.g. F, t, r) with confidence intervals, effect sizes, degrees of freedom and P value noted<br>*Give P values as exact values whenever suitable.*
☒	☐	For Bayesian analysis, information on the choice of priors and Markov chain Monte Carlo settings
☐	☒	For hierarchical and complex designs, identification of the appropriate level for tests and full reporting of outcomes
☒	☐	Estimates of effect sizes (e.g. Cohen's d, Pearson's r), indicating how they were calculated

Our web collection on statistics for biologists contains articles on many of the points above.

Software and code

Policy information about availability of computer code

Data collection	Data were made available from previous studies. No software was used for data collection.
Data analysis	Processing and analysis code: https://github.com/garedaba/micro-brain Zenodo: 10.5281/zenodo.13917290<br>Python 3.7, Tensorflow 2.4.1, antspyx 0.2.7, Freesurfer 7.3.2, OpenCV 4.5.2, MightyMosaic 1.2.3, MSM [https://github.com/ecr05/MSM_HOCR], statsmodels 0.13.5, scikit-learn 0.24.2

For manuscripts utilizing custom algorithms or software that are central to the research but not yet described in published literature, software must be made available to editors and reviewers. We strongly encourage code deposition in a community repository (e.g. GitHub). See the Nature Portfolio guidelines for submitting code & software for further information.

Data

Policy information about availability of data

All manuscripts must include a data availability statement. This statement should provide the following information, where applicable:

- Accession codes, unique identifiers, or web links for publicly available datasets
- A description of any restrictions on data availability
- For clinical datasets or third party data, please ensure that the statement adheres to our policy

The µBrain digital template with corresponding cortical surfaces, atlas labels and processed microarray data used in this study is available from https://garedaba.github.io/micro-brain and is deposited at Zenodo: 10.5281/zenodo.10622336. Source data are provided with this paper.

All dHCP data, fetal brain reconstructions, brain region segmentations and cortical surfaces are available for download from the NDA https://nda.nih.gov/edit_collection.html?id=3955. Source histological and microarray data are available from the Allen Brain Institute https://www.brainspan.org/

Research involving human participants, their data, or biological material

Policy information about studies with human participants or human data. See also policy information about sex, gender (identity/presentation), and sexual orientation and race, ethnicity and racism.

Reporting on sex and gender	For MRI analyses, after image processing and quality control, the final dataset comprised n=195 fetal MRI datasets acquired from n=190 fetuses (88 female). Scaling models were repeated including sex as a covariate to test the effect of sex on cortical scaling. The inclusion of sex and sex:age interaction effects in the scaling model did not affect estimated vertex scaling coefficients<br><br>For gene expression analysis, sex was not considered due to limited number of specimens available (3 female, 1 male).
Reporting on race, ethnicity, or other socially relevant groupings	n/a
Population characteristics	After image processing and quality control, the final dataset comprised n=195 fetal MRI datasets acquired from n=190 fetuses aged 21+1 to 38+2 gestational weeks (88 female).
Recruitment	Fetal MRI datasets (n=240 scans from 229 fetuses aged between 21+1 and 38+2 gestational weeks+days) were acquired as part of the Developing Human Connectome Project (dHCP).
Ethics oversight	The dHCP study was approved by the UK Health Research Authority (Research Ethics Committee reference 452 number: 14/LO/1169) and written parental consent was obtained in every case for imaging and open data release of the anonymized data.

Note that full information on the approval of the study protocol must also be provided in the manuscript.

Field-specific reporting

Please select the one below that is the best fit for your research. If you are not sure, read the appropriate sections before making your selection.

- [x] Life sciences
- [ ] Behavioural & social sciences
- [ ] Ecological, evolutionary & environmental sciences

For a reference copy of the document with all sections, see nature.com/documents/nr-reporting-summary-flat.pdf

Life sciences study design

All studies must disclose on these points even when the disclosure is negative.

Sample size	No sample size calculations were performed. Sample size was based on recruitment inclusion and exclusion criteria of the dHCP.
Data exclusions	MRI data were excluded based on visual inspection of tissue segmentations. Prior to analysis, vertexwise outliers were identified and removed. To account for age-related increases in area, outliers were identified using a sliding window over age (outliers >2.5 S.D. from the mean within a given window, maximum window size=25 scans, sorted by age). Data from five scans were removed prior to analysis due to the presence of outliers in more than 5% of vertices.<br><br>For microarray data, low signal probes designated 'absent' were removed (34.67% of probes), as were tissue samples from the marginal zone, subpial granular zone and subcortical and midbrain structures (54.46% of samples). Where multiple probes mapped to a single gene, the probe with the highest differential stability (DS) the average pairwise correlation between tissue sample expression over all specimens, was assigned. Probes with DS<0.2 were removed. Any probes with missing data in more than 10% of tissue samples were removed (n=1253).
Replication	Sensitivity analyses were performed to determine robustness of scaling models. Independent data were used to validate findings where available.
Randomization	n/a - observational study
Blinding	n/a - observational study

Reporting for specific materials, systems and methods

We require information from authors about some types of materials, experimental systems and methods used in many studies. Here, indicate whether each material, system or method listed is relevant to your study. If you are not sure if a list item applies to your research, read the appropriate section before selecting a response.

Materials & experimental systems

n/a	Involved in the study
☒	☐ Antibodies
☒	☐ Eukaryotic cell lines
☒	☐ Palaeontology and archaeology
☒	☐ Animals and other organisms
☒	☐ Clinical data
☒	☐ Dual use research of concern
☒	☐ Plants

Methods

n/a	Involved in the study
☒	☐ ChIP-seq
☒	☐ Flow cytometry
☐	☒ MRI-based neuroimaging

Plants

Seed stocks: *Report on the source of all seed stocks or other plant material used. If applicable, state the seed stock centre and catalogue number. If plant specimens were collected from the field, describe the collection location, date and sampling procedures.*

Novel plant genotypes: *Describe the methods by which all novel plant genotypes were produced. This includes those generated by transgenic approaches, gene editing, chemical/radiation-based mutagenesis and hybridization. For transgenic lines, describe the transformation method, the number of independent lines analyzed and the generation upon which experiments were performed. For gene-edited lines, describe the editor used, the endogenous sequence targeted for editing, the targeting guide RNA sequence (if applicable) and how the editor was applied.*

Authentication: *Describe any authentication procedures for each seed stock used or novel genotype generated. Describe any experiments used to assess the effect of a mutation and, where applicable, how potential secondary effects (e.g. second site T-DNA insertions, mosiacism, off-target gene editing) were examined.*

Magnetic resonance imaging

Experimental design

Design type: n/a - structural MRI only

Design specifications: n/a

Behavioral performance measures: n/a

Acquisition

Imaging type(s): Structural

Field strength: 3T

Sequence & imaging parameters: Fetal MRI datasets (n=240 scans from 229 fetuses aged between 21+1 and 38+2 gestational weeks+days) were acquired as part of the Developing Human Connectome Project (dHCP) using a Philips Achieva 3T system, with a 32-channel cardiac coil in maternal supine position. Structural T1-weighted (T1w), T2w, functional MRI and diffusion MRI data were acquired for a total scan time of approximately 45 minutes.85 T2-weighted SSTSE volumes were acquired with TE=250ms, acquisition resolution 1.1 x 1.1mm, slice thickness 2.2mm, -1.1mm gap and 6 stacks. All 3D brain images were reconstructed using a fully automated slice-to-volume reconstruction (SVR) pipeline86 to 0.5mm resolution and reoriented to the standard radiological space.

Area of acquisition: Whole brain

Diffusion MRI: ☐ Used ☒ Not used

Preprocessing

Preprocessing software: We used an optimised neonatal tissue segmentation pipeline (Draw-EM) with tissue priors adapted to a fetal MRI template to create a ‘first-pass’ tissue segmentation for each fetal MRI volume. Tissue segmentations were then visually checked and extensive manual corrections performed where needed to correct gross segmentation errors and ensure accuracy of tissue boundaries (CSF/cortex/white matter). Manually-corrected tissue segmentations were then used to generate anatomically and topologically correct inner and outer cortical surfaces using Deformable. Note that all intensity-based correction terms were turned off during surface reconstruction and each surface was generated using just the corrected tissue segmentations. At each stage, images and derived outputs were visually inspected for accuracy.

Normalization: Nonlinear surface registration with multimodal surface matching

Normalization template: dHCP surface template: https://doi.gin.g-node.org/10.12751/g-node.qj5hs7/

Noise and artifact removal	n/a (only structural MRI was used - extensive manual correction was performed to ensure correct tissue segmentation)
Volume censoring	n/a (only structural MRI was used)

Statistical modeling & inference

Model type and settings	For MRI data, at each vertex, we modeled scaling relationships with brain size by estimating the log-log regression coefficient for total surface area as a predictor of vertex area: log(vertex_area) ~ 1 + log(total_area) + e. Models were repeated excluding repeated measures (n=5) to satisfy OLS assumptions of independence<br><br>For microarray data: For each gene (n=8771), we modelled the main effects of cortical tissue zone, region and timepoint on expression using a general linear model. Significant effects (p<0.01) were identified after False Discovery Rate correction for multiple comparisons over genes.
Effect(s) tested	n/a

Specify type of analysis: ☐ Whole brain ☐ ROI-based ☒ Both

Anatomical location(s)	Histologically-defined cortical parcellation
Statistic type for inference<br>(See Eklund et al. 2016)	n/a - vertexwise maps of areal scaling (β coefficients) were parcellated using the µBrain cortical labels, calculating average scaling within each parcel for further analysis.
Correction	n/a

Models & analysis

n/a	Involved in the study
☒	☐ Functional and/or effective connectivity
☒	☐ Graph analysis
☐	☒ Multivariate modeling or predictive analysis

Multivariate modeling and predictive analysis	PCA was applied to microarray data.
